# Bardoxolone Methyl: A Comprehensive Review of Its Role as a Nrf2 Activator in Anticancer Therapeutic Applications

**DOI:** 10.3390/ph18070966

**Published:** 2025-06-27

**Authors:** Valentina Schiavoni, Tiziana Di Crescenzo, Valentina Membrino, Sonila Alia, Sonia Fantone, Eleonora Salvolini, Arianna Vignini

**Affiliations:** 1 Department of Clinical Sciences, Polytechnic University of Marche, 60100 Ancona, Italy; v.schiavoni@pm.univpm.it (V.S.); t.dicrescenzo@pm.univpm.it (T.D.C.); v.membrino@pm.univpm.it (V.M.); s.alia@pm.univpm.it (S.A.); 2 Scientific Direction, IRCCS INRCA, 60124 Ancona, Italy; s.fantone@inrca.it; 3 Research Center of Health Education and Health Promotion, Polytechnic University of Marche, 60100 Ancona, Italy

**Keywords:** bardoxolone methyl, CDDO-Me, RTA 402, triterpenoid

## Abstract

Bardoxolone methyl, also known as CDDO-Me or RTA 402, is a synthetic oleanane triterpenoid that has garnered significant attention as a potent pharmacological activator of the nuclear factor erythroid 2-related factor 2 (Nrf2) pathway. Nrf2 is a master regulator of cellular redox homeostasis, controlling the expression of genes involved in antioxidant defense, detoxification, and mitochondrial function. By inducing Nrf2 and promoting the transcription of downstream antioxidant response element (ARE)-driven genes, bardoxolone methyl enhances cellular resilience to oxidative stress and inflammation. This mechanism is central not only to its cytoprotective effects but also to its emerging role in oncology. A number of studies investigated the effects of bardoxolone methyl in several malignancies including breast cancer, lung cancer, pancreatic ductal adenocarcinoma, prostate cancer, colorectal cancer, oral and esophageal squamous cell carcinoma, ovarian cancer and glioblastoma. Studies in the literature indicate that bardoxolone methyl exhibits anticancer activity through several mechanisms, including the suppression of cell proliferation, induction of cell cycle arrest and apoptosis, inhibition of epithelial–mesenchymal transition (EMT), and impairment of cancer cell stemness. Additionally, bardoxolone methyl modulates mitochondrial function, reduces glycolytic and oxidative phosphorylation capacities, and induces reactive oxygen species (ROS)-mediated stress responses. In this review, we summarize the available literature regarding the studies which investigated the effects of bardoxolone methyl as anticancer agent.

## 1. Introduction

**Figure 1:**
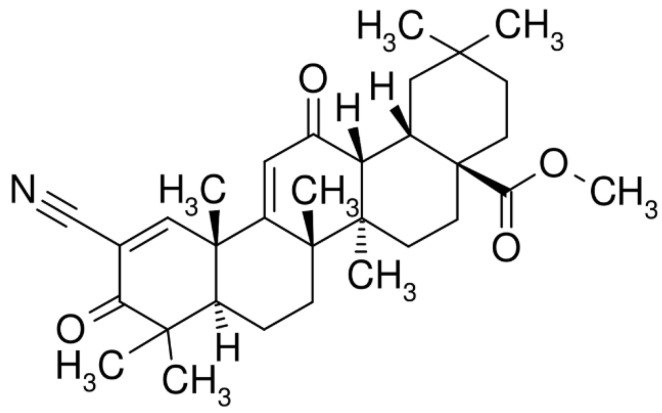
Chemical structure of methyl-2-cyano-3,12-dioxooleana-1,9(11)-dien-28-oate (bardoxolone methyl).

Natural compounds are bioactive molecules that can be found in a variety of plants, fungi and bacteria, and are widely used in natural medicine for their anti-inflammatory, antioxidant and anticancer properties [1,2,3,4,5]. Triterpenoids represent a variegate class of bioactive plant-derived molecules, where they are biosynthesized via the cyclization of squalene [6]. Naturally occurring triterpenoids like oleanolic acid and ursolic acid possess only limited anti-inflammatory, antiviral and anticancer effects. To improve their therapeutic efficacy, numerous synthetic derivatives of oleanolic acid have been developed [7,8,9,10,11]. Among these, 2-cyano-3,12-dioxooleana-1,9-dien-28-oic acid (CDDO) and its derivatives—most notably the methyl ester form known as bardoxolone methyl—were originally synthesized for the treatment and prevention of cancer and inflammatory conditions [12,13,14]. The molecular structure of bardoxolone methyl is shown in Figure 1. These synthetic triterpenoids act as powerful inhibitors of the de novo synthesis of pro-inflammatory enzymes, including inducible nitric oxide synthase (iNOS) and cyclooxygenase 2 (COX-2) [15]. Nonetheless, bardoxolone methyl demonstrated enhanced potency compared to CDDO in several contexts, including cancer treatment or cancer-preventive activity [16].

## 2. Bardoxolone Methyl: Pharmacokinetic Properties and Biological Mechanisms

Owing to the limited oral bioavailability of the parent compound bardoxolone, structurally modified derivatives such as bardoxolone methyl were synthesized to enhance systemic absorption following oral administration. However, despite these modifications, the crystalline, unsolvated form of bardoxolone methyl still exhibited suboptimal bioavailability. To address this limitation, an amorphous spray-dried dispersion (SDD) formulation was developed and employed in the Phase III BEACON trial to assess the efficacy of bardoxolone methyl in patients with chronic kidney disease (CKD) [17]. In a separate pharmacokinetic evaluation in healthy individuals, administration of 30 mg of the SDD formulation resulted in higher systemic bioavailability compared to 150 mg of the crystalline counterpart, while maintaining a comparable exposure profile.

To support clinical development, Hong et al. established a rapid liquid chromatography–tandem mass spectrometry (LC-MS/MS) method for quantifying plasma concentrations of bardoxolone methyl in a Phase I trial involving 47 patients with advanced, treatment-resistant solid tumors and lymphoid malignancies [18]. The objectives of this early-phase trial were to identify dose-limiting toxicities (DLTs), determine the maximum tolerated dose (MTD), and establish a recommended dosing regimen for subsequent Phase II investigations. Additional goals included assessment of both single-dose and steady-state pharmacokinetic parameters, as well as preliminary evaluation of anti-neoplastic activity. Patients received oral SDD bardoxolone methyl once daily for 21 consecutive days within a 28-day treatment cycle. The initial dose was set at 5 mg, approximately one-tenth of the dose associated with significant toxicity in preclinical rodent models. Serial blood sampling was performed prior to and at multiple timepoints (up to 24 h) following administration of both the first and final doses of cycle one. Pharmacokinetic analysis revealed that bardoxolone methyl exhibited slow and saturable oral absorption kinetics, with a prolonged terminal elimination half-life (mean 39 ± 20 h at 900 mg/day, *n* = 19), nonlinear dose–exposure relationships at higher dose levels (600–1300 mg/day), and considerable inter-individual variability.

At the 900 mg dosage, the median time to reach maximum plasma concentration (T max) was 4.1 ± 3.4 h. The average steady-state trough and peak plasma levels were 8.8 ± 4.3 ng/mL and 24.7 ± 13.3 ng/mL, respectively, resulting in an approximate peak-to-trough ratio of 2.8 ± 1.6. This suggests that once-daily administration can achieve relatively consistent plasma exposure in individual patients. Interpatient variability in pharmacokinetic parameters was notably high, ranging from 64–77% after the first dose and 39–54% after the final dose at the 900 mg/day level. Oral clearance appeared to increase with dose, whereas terminal half-life remained dose-independent, implying a saturable absorption mechanism. The prolonged elimination half-life supports the feasibility of once-daily dosing in future clinical studies [19].

## 3. Targets of Bardoxolone Methyl

**Figure 2:**
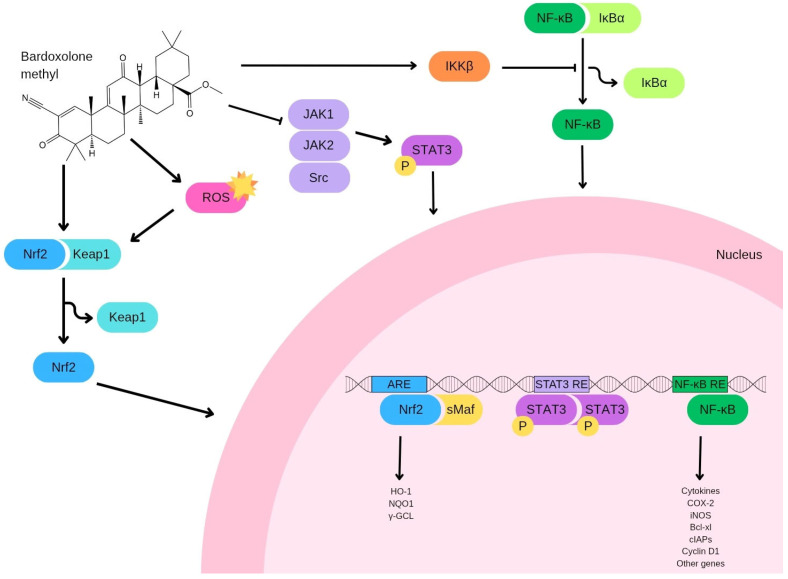
Mechanisms involved in bardoxolone methyl effects.

**Figure 3:**
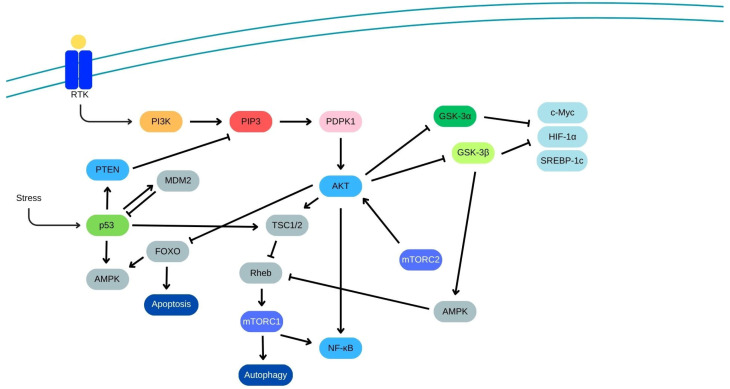
The PI3K-Akt-mTOR pathway with autophagy and apoptosis regulation.

Many natural bioactive molecules are known to exerts their activity through the modulation of oxidative stress [20,21,22,23]. Bardoxolone methyl, a synthetic derivative of triterpenoids, is widely recognized for its potent ability to activate the nuclear factor erythroid 2-related factor 2 (Nrf2). This transcription factor, consisting of 605 amino acids, plays a central role in n managing the cellular defense response against oxidative stress, a factor implicated in the development of both malignant and non-malignant diseases [24,25,26,27,28,29,30,31,32,33]. Upon activation, Nrf2 promotes the transcription of genes involved protecting cells from damage induced by reactive oxygen species (ROS) [34,35]. It belongs to the basic leucine zipper (bZip) family of transcription factors and exert its regulatory effects by binding to specific DNA sequences. The activity of Nrf2 is tightly regulated by the Kelch-like ECH-associated protein 1 (Keap1), which acts as a negative regulator by facilitating Nrf2 degradation through the proteasomal pathway (Figure 2) [36,37]. This regulation occurs through the formation of a complex involving Keap1 and an E3 ubiquitin ligase complex composed of Cullin3 (Cul3) and Ring-box 1 (Rbx1). Additionally, Nrf2 may also be targeted for degradation through a Keap1-independent mechanism involving glycogen synthase kinase 3 (GSK3) and β-TrCP (F-box/WD repeat-containing protein 1A) [38]. In this alternative pathway, inactivation of Akt allows GSK3 to phosphorylate Nrf2, which in turn marks it for recognition by β-TrCP, leading to its degradation by the proteasome [39]. Several other kinases, such as AMP-activated protein kinase (AMPK), members of the mitogen-activated protein kinase (MAPK) family, and the mechanistic target of rapamycin complex 1 (mTORC1), are also known to influence Nrf2 activity, further modulating its cellular functions (Figure 3). Under conditions of oxidative stress, Keap1 undergoes oxidation at its cysteine residues—specifically Cys151, Cys226, Cys613, and Cys624—by electrophilic compounds and oxidants [40]. This modification induces structural changes in Keap1, including the formation of intramolecular disulfide bridges, which disrupt its interaction with Nrf2. Consequently, Keap1 undergoes degradation via the proteasome, permitting the stabilization and nuclear accumulation of Nrf2. Once translocated into the nucleus, Nrf2 forms heterodimers with musculoaponeurotic fibrosarcoma (Maf) proteins and binds to antioxidant response elements (AREs) in DNA, promoting the transcription of genes involved in antioxidative and detoxification processes. An alternative pathway for Keap1 degradation has also been identified, which involves the sequestration of Keap1 by p62, a protein that activates autophagy [41]. This process plays a critical role in maintaining the turnover of the Keap1-Nrf2 system and ensures the proper regulation of Nrf2 activity. Within the nucleus, Nrf2 regulates the expression of various antioxidant and detoxifying enzymes, such as glutamate-cysteine ligase modifier (GCLM) subunits, heme oxygenase-1 (HO-1), NADPH dehydrogenase quinone 1 (NQO1), glutathione S-transferase (GST), glutathione peroxidase (GPx), and glutamate-cysteine ligase catalytic (GCLC) [42]. One of the primary ways bardoxolone methyl exerts its protective effects is through activation of the Keap1/Nrf2/ARE pathway, a critical axis involved in cellular resistance to oxidative and electrophilic stress (Figure 2) [43].

Bardoxolone methyl exerts its anticancer effects through both Nrf2-dependent and Nrf2-independent mechanisms. The Nrf2-dependent pathway primarily involves the activation of the Keap1-Nrf2-ARE signaling cascade, leading to the upregulation of cytoprotective genes involved in antioxidant defense, detoxification, and metabolic regulation. This can reduce oxidative stress and inflammation, contributing to tumor suppression in certain contexts [44]. However, bardoxolone methyl also displays Nrf2-independent effects, including the direct inhibition of IκB kinase (IKK) and subsequent suppression of NF-κB signaling, which reduces pro-inflammatory cytokine production and can impair cancer cell survival. Additionally, bardoxolone methyl has been shown to modulate mitochondrial function, induce apoptosis, and affect cellular metabolism independently of Nrf2 activation [45,46].

Inflammation is an important biological process the body use as defense against harmful stimuli, such as pathogens. However, this process is also involved in the onset and progression of several diseases including cancer [47,48,49,50,51,52]. The NF-κB signaling pathway plays a key role in inflammation and represents another critical target of bardoxolone methyl. NF-κB activates several genes that drive inflammation, cellular proliferation, and survival. Given its role in regulating the inflammatory microenvironment, this pathway can clearly contribute to tumorigenesis. It is proposed that there is cross-talk between the Nrf2 and NF-κB signaling pathways, allowing them to jointly influence the expression and activity of downstream target proteins. This theory is reinforced by multiple studies showing that elements of each pathway can either activate or suppress components of the other, both directly and indirectly. Under conditions of oxidative stress, IKK is activated, leading to the phosphorylation of IκB. This modification triggers the dissociation and subsequent nuclear translocation of NF-κB and, once in the nucleus, NF-κB facilitates the transcription of pro-inflammatory mediators, including IL-6, TNF-α, iNOS, IL-1, and COX-2 [53]. Thus, bardoxolone methyl directly blocks the NF-κB pathway by inhibiting IKK [54].

It has been reported that Nrf2 can bind SIRT3 thus activated Nrf2 upregulates SIRT3 expression and activity, by improving mitochondrial function [55]. SIRT3, a mitochondrial NAD^+^-dependent deacetylase, plays a significant role in regulating epigenetic mechanisms through its impact on cellular metabolism and redox homeostasis. By modulating the activity of key metabolic enzymes, SIRT3 influences the availability of critical metabolites such as acetyl-CoA, α-ketoglutarate, and NAD^+^—all of which serve as essential cofactors for epigenetic modifiers including histone acetyltransferases, histone demethylases, and sirtuins. Additionally, SIRT3-mediated control of mitochondrial function and reactive oxygen species (ROS) levels can indirectly affect the chromatin landscape, thereby influencing gene expression. Through these mechanisms, SIRT3 links mitochondrial metabolic status to epigenetic regulation and transcriptional adaptation in response to physiological and environmental stimuli. Therefore, it is conceivable to hypothesize that bardoxolone methyl might impact the epigenetics through the Nrf2/SIRT3 pathway.

Nrf2 is regulated also through dynamic post-translational modifications (PTMs) and epigenetic mechanisms that fine-tune its antioxidant and cytoprotective functions. PTMs critically modulate Nrf2 stability, activity, and localization, with ubiquitination serving as the primary degradation signal through Keap1-mediated proteasomal targeting under basal conditions. Phosphorylation by kinases such as PKC enhances Nrf2 stability by disrupting Keap1 binding, while acetylation and deubiquitination (e.g., via CYLD) further regulate its nuclear accumulation and transcriptional activity. Epigenetic regulation involves DNA methylation at the Nrf2 promoter, which suppresses its expression, and histone modifications like acetylation or methylation that alter chromatin accessibility. Long non-coding RNAs (MALAT1, PVT1, mir4435-2HG, TUG1) interact with Nrf2 to either stabilize its mRNA or modulate its transcriptional network. These epigenetic mechanisms synergize with PTMs to control Nrf2-driven responses to oxidative stress, inflammation, and metabolic demands. For instance, stress-induced histone acetylation facilitates Nrf2 binding to antioxidant response elements, while site-specific phosphorylation determines its nuclear translocation efficiency. The integration of these layered regulatory processes enables precise spatiotemporal control of Nrf2 activity, balancing cytoprotection against potential oncogenic effects from sustained activation [56].

Under physiological conditions, Nrf2 activation confers cytoprotection by inducing the expression of antioxidant and detoxification genes, thereby maintaining redox homeostasis and preventing DNA damage. However, in several malignancies, persistent or aberrant activation of Nrf2—often due to mutations in its negative regulator KEAP1—can contribute to tumor cell survival, proliferation, and resistance to chemotherapy and radiotherapy [57,58]. This sustained Nrf2 activity promotes metabolic reprogramming by enhancing the pentose phosphate pathway, glutathione biosynthesis, and NADPH production, thereby supporting the anabolic and antioxidant needs of rapidly growing tumor cells [59]. Thus, while Nrf2 is critical for normal cellular defense, its dysregulation can facilitate oncogenesis and therapy resistance, highlighting its dualistic role in cancer biology.

Signal Transducers and Activators of Transcription (STATs) are essential transcription factors that regulate key cellular functions, including transformation, proliferation, survival, invasion, and metastasis. Among them, STAT3 is notably constitutively activated in a wide range of cancers [60,61]. Activation typically begins when ligands, such as interleukin-6 (IL-6), bind to their corresponding growth factor receptors, triggering Janus Kinases (JAKs) to phosphorylate the receptors. This phosphorylation event prompts STAT proteins to dimerize, translocate into the nucleus, and initiate transcription of several genes, such as cyclin D1, myc, and survivin [62]. Bardoxolone methyl exerts its effect by directly interacting with Cys1077 on JAK1, effectively inhibiting JAK1 phosphorylation and preventing the activation of STAT3, thus hindering STAT3 dimerization. Additionally, since both JAK and Src kinases can activate STAT3, CDDO-Me can block STAT3 activation through the inhibition of either JAK2 or Src [63].

## 4. Bardoxolone Methyl and Cancer

### 4.1. Lung Cancer

Liby et al. reported that bardoxolone methyl is a highly effective inhibitor of lung carcinogenesis in A/J mice. In this study, female A/J mice were treated with vinyl carbamate, a highly mutagenic carcinogen which is able to generate lung adenocarcinoma within 16 weeks. When these mice were administered Bardoxolone methyl starting one week after carcinogen exposure, it was detected a significant reduction in the number and size of lung tumors in vivo. Specifically, treatment with Bardoxolone methyl resulted in 92% reduction in average tumor burden compared to controls. Furthermore, bardoxolone methyl has a great impact also on the aggressiveness of tumors since only one high-grade tumor was observed in the mice treated with the drug, while 36% of the control tumors were histologically classified as high grade [64]. The anticarcinogenic effects of bardoxolone methyl were linked to its anti-inflammatory effects (i.e., induction of HO-1 through the Nrf2 pathway, suppression of STAT phosphorylation, and inhibition of iNOS).

### 4.2. Breast Cancer

Several studies investigated the effect of bardoxolone methyl in cellular and in vivo models of breast cancer, which is the most common malignancy in women [65]. Liby et al. investigated the potential anticarcinogenic effect against estrogen receptor (ER)-negative breast tumors (ER^-^) of bardoxolone methyl and the rexinoid LG100268 using mouse mammary tumor virus-neu transgenic model. Mice were fed a control diet or diets supplemented with bardoxolone methyl (100 mg/kg diet), LG100268 (60 mg/kg diet), or both compounds in combination. Treatment with bardoxolone methyl and LG100268 significantly delayed the onset of ER^-^ tumors, with delays of 14 and 24 weeks, respectively, in the time required to reach a 50% tumor incidence compared to the control group. Moreover, the combination of bardoxolone methyl and LG100268 proved to be substantially more effective compared to single drug administration, as only one tumor was observed in the combination group after 45 weeks, whereas all control mice had developed tumors by that time [66]. Furthermore, the same group investigated the effects of bardoxolone methyl, LG100268, and their combination in LSL-KrasG12D/+, LSL-Trp53R127H/+, Pdx-1-Cre (KPC) mouse model of pancreatic cancer. Starting at 4 weeks of age, mice were administered either a powdered control diet or a diet supplemented with bardoxolone methyl, LG100268, or a combination of these molecules, and continued on these diets until they developed visible symptoms of pancreatic cancer. Treatment with bardoxolone methyl, LG100268, or their combination, significantly prolonged survival of mice by 3 to 4 weeks [67]. This result was particularly significant since this KPC model of pancreatic cancer is extremely aggressive with an average survival time of only 20.5 ± 0.9 weeks for controls. Among the possible involved pathways, authors highlighted the increased the STAT3 signaling, which is well known to regulate in a reciprocal way the Nrf2 signaling [68]. Tran et al. investigated the pharmacological effects of bardoxolone methyl in a pertinent model of ER^-^ breast cancer, specifically the polyoma-middle T (PyMT) mouse model, where the oncoprotein plays a central role in driving carcinogenesis. The treatment regimen involved administering bardoxolone methyl to mice beginning at 4 weeks of age. The results of this study showed that bardoxolone methyl notably delayed the initiation of tumor formation, increasing the age at which the first tumor appeared by an average of 4.3 weeks, and extended overall survival by 5.2 weeks. Furthermore, bardoxolone methyl was found to suppress the infiltration of tumor-associated macrophages into the mammary tissue of PyMT mice at 12 weeks of age. The treatment also led to a reduction in the levels of the chemokines CXCL12 and CCL2 in the primary tumor cells of PyMT mice and reduced the secretion of matrix metalloproteinase-9 (MMP-9) from primary tumor cells and inhibited their proliferation, which was associated with a downregulation of cyclin D1 expression, as well as a decrease in phosphorylation of the epidermal growth factor receptor (EGFR) and signal transducer and STAT3 [69]. Kim et al. investigated the effect of bardoxolone methyl administration in a murine model where the BRCA1 gene is deleted in combination with a mutation in one allele of the p53 tumor suppressor gene. Starting at 12 weeks of age, Brca1^Co/Co^; MMTVCre; p53^+/−^ mice were administered either a control diet or a diet supplemented with bardoxolone methyl. The administration of bardoxolone methyl resulted in a notable delay in tumor development, extending the latency period by an average of 5.2 weeks in the BRCA1-deficient mice. Moreover, it was observed that the levels of ErbB2, phosphorylated ErbB2 (p-ErbB2), and cyclin D1 progressively increased over time in the mammary glands of BRCA1-deficient mice and the treatment with bardoxolone methyl inhibited the persistent phosphorylation of ErbB2 in the tumor tissues of these mice. Experiments performed on the BRCA1 mutant cell line W780 showed that bardoxolone methyl directly interacted with ErbB2, reducing its constitutive phosphorylation, suppressing cell proliferation, and inducing a G0/G1 cell cycle arrest [70]. These findings suggest that bardoxolone methyl holds promise as a therapeutic agent for the prevention of breast cancer associated with BRCA1 mutations. Ling et al. investigated the effect of bardoxolone methyl in murine 4T1 highly chemoresistant breast tumor model. In vitro, treatment of 4T1 cells with 500 nmol/L bardoxolone methyl for 2 h resulted in STAT3 inactivation, inactivation of Src and Akt, a decrease in c-Myc levels, blockade of the cell cycle in G2-M phase, suppression of invasive growth of 4T1 cells, and no induction of apoptosis. In vivo, bardoxolone methyl treatment initiated 1 day after tumor implantation completely eradicated 4T1 breast cancer growth and lung metastases in mice, and significantly inhibited tumor progression when treatment began 5 days post-implantation, suggesting that bardoxolone methyl may offer therapeutic potential in breast cancer treatment, potentially through the inactivation of STAT3 signaling [71]. Jeong et al. demonstrated that exposure to bardoxolone methyl induced extensive vacuolization originating from the endoplasmic reticulum in multiple breast carcinoma cell lines which results in apoptotic death. The vacuolization was strongly associated with increased cytosolic calcium concentrations, primarily due to influx from the extracellular environment, and enhanced the intracellular production of ROS. Furthermore, bardoxolone methyl treatment resulted in a rapid depletion of intracellular levels of the anti-apoptotic protein c-FLIPL, thus suggesting that bardoxolone methyl exerts its cytotoxic effects on breast cancer cells via dual mechanisms: ER dysfunction mediated by Ca^2+^/ROS signaling, and caspase-dependent apoptosis driven by c-FLIPL suppression [72].

Angiogenesis is an important and tightly regulated process involved in the migration, growth, and differentiation of endothelial cells, and plays a key role in several diseases including cancer [73,74,75]. Ball et al. explored the effect of bardoxolone methyl administration on breast cancer tumor microenvironment. Bardoxolone methyl treatment in four-week-old female PyMT mice resulted in a significant downregulation of immunosuppressive and proangiogenic mediators such as interleukin-10 (IL-10) and vascular endothelial growth factor (VEGF), while enhanced tumor necrosis factor (TNF) production. Moreover, bardoxolone methyl reduced surface expression of CD206 and CD115 markers which characterize an alternatively activated immunosuppressive macrophage phenotype. Transcriptional profiling of tumor-associated macrophages isolated from treated tumors revealed an upregulation of gene networks linked to immune activation and a concomitant decrease in CCL2 expression, consistent with reduced infiltration of tumor-associated macrophages into the tumor bulk. Immune phenotyping of lymphoid compartments revealed that bardoxolone methyl administration led to a relative depletion of CD4^+^ T cells in the spleen, accompanied by a marked enrichment of CD8^+^ T cells within both the spleen and tumor microenvironment. Furthermore, a significant reduction in intratumoral CD4^+^Foxp3^+^ regulatory T cells (Tregs) was observed, suggesting that bardoxolone methyl disrupts immunosuppressive regulatory networks within the tumor microenvironment. Thus, authors concluded that bardoxolone methyl effectively alleviates immune suppression in the breast cancer microenvironment, thereby enhancing host antitumor adaptive immunity [76]. El-Ashmawy et al. evaluated the radioprotective potential of bardoxolone methyl in vitro using a diverse panel of human epithelial cell lines derived from breast and lung tissues including normal, premalignant, and cancer phenotypes. The cells were subjected to γ-irradiation with or without pre-treatment with bardoxolone methyl administered approximately 18 h prior to exposure. In non-malignant epithelial populations, bardoxolone methyl significantly attenuated radiation-induced cytotoxicity, as indicated by an average dose-modifying factor (DMF) of 1.3. This protective effect was abrogated upon Nrf2 inhibition, higlighting the essential role of this transcription factor in mediating the compound’s radioprotective mechanism. Notably, bardoxolone methyl failed to confer radioprotection in fully transformed cancer cell lines or in human bronchial epithelial cells subjected to sequential oncogenic alterations, suggesting specificity toward non-transformed cellular contexts. Moreover, bardoxolone methyl effectively mitigated DNA damage in irradiated human lymphocytes, further demonstrating its cytoprotective capacity. These findings suggest a possible therapeutic application of bardoxolone methyl which could selectively enhance the resilience of normal tissues to ionizing radiation via Nrf2 activation, without safeguarding tumor cells, making it a promising non-toxic drug which could be an adjunct in radiation oncology to minimize treatment-associated toxicity and improve clinical outcomes [77]. Zhou et al. reported that bardoxolone methyl promotes ubiquitination and proteolytic degradation via a lysosomal pathway of lipoprotein receptor-related protein 6 (LRP6) and Frizzled-7 (FZD7). In breast cancer cell models, bardoxolone methyl treatment led to significant depletion of phosphorylated LRP6 and phosphorylated Disheveled 2 (DVL2), alongside suppression of cytoplasmic β-catenin activation, resulting in the downregulation of Wnt target genes and cancer stem cell (CSC) markers [78]. This effect is particularly important since cancer stem cells are considered a major reason for therapy failure and recurrence [79]. In vivo efficacy was demonstrated using a transgenic murine model bearing mammary tumors driven by the mouse mammary tumor virus (MMTV)-Wnt1 oncogene since bardoxolone methyl administration markedly impaired tumor progression, reduced expression of phosphorylated and total LRP6, phosphorylated and unphosphorylated DVL2, active β-catenin, and downstream Wnt-responsive and CSC-associated transcripts. Thus, taken together, these findings suggest that bardoxolone methyl is a strong Wnt/β-catenin signaling inhibitor that could be a promising bioactive molecule against breast cancer [78]. Refaat et al. investigated the impact of bardoxolone methyl on cellular migration, proliferation, and mitochondrial bioenergetics in the MCF7 human breast cancer cell line. Bardoxolone methyl treatment resulted in a time-dependent decrease in cell migration, coupled with a decline in mitochondrial respiratory function and these inhibitory effects on migration and mitochondrial activity were amplified in the presence of exogenous fatty acids. Gene expression analyses confirmed that bardoxolone methyl modulates Nrf2 target transcripts, including GCLC (glutamate-cysteine ligase catalytic subunit) and UCP1 (uncoupling protein 1), indicating transcriptional engagement of oxidative stress and metabolic pathways. After 24 h of exposure, bardoxolone methyl significantly reduced glycolytic reserve and oxidative phosphorylation capacity, while elevated mitochondrial ROS levels and depleted intracellular glutathione pools. Importantly, co-treatment with NAC mitigated the bardoxolone methyl-induced mitochondrial dysfunction, suggesting ROS as a key mediator. Moreover, bardoxolone methyl promoted phosphorylation of AKT, triggered DNA damage, and inhibited cellular proliferation. Inhibition of the proteasome abrogated bardoxolone methyl-dependent changes in p65 phosphorylation and SOD2 expression, implying a role for non-canonical NF-κB signaling in bardoxolone methyl downstream effects [80].

### 4.3. Prostate Cancer

Prostate cancer is a biologically heterogeneous disease and the second most common cancer in males (the fifth leading cause of cancer mortality) [81,82]. Prostate cancer is characterized different histopathological subtypes but the most common is adenocarcinoma of the prostate [83,84]. Deeb et al. investigated the effect of bardoxolone methyl against prostate adenocarcinoma in a murine model. Drug administration effectively inhibited the progression of preneoplastic lesions, including both low- and high-grade prostate intraepithelial neoplasms, to invasive adenocarcinoma in over 70% of the mice, with no significant toxicity observed [85,86]. Notably, further investigations by the same group also revealed that bardoxolone methyl treatment suppressed the metastatic process. The therapeutic effects of bardoxolone methyl were linked to a reduction in proteins involved in the regulation of telomerase reverse transcriptase (TERT) production and phosphorylation, suggesting that telomerase is a potential target of bardoxolone methyl for the prevention and treatment of prostate cancer [87]. The same research group examined the effects of bardoxolone methyl on both hormone-sensitive (LNCaP) and hormone-refractory (PC-3 and DU145) human prostate cancer cell lines revealing that bardoxolone methyl effectively inhibited the growth of prostate cancer cells. A detailed investigation into the antitumor mechanisms of bardoxolone methyl clarified that it induced apoptosis in LNCaP and PC-3 cell lines by activating caspases-3, -8, and -9, disrupting mitochondrial integrity, and inhibiting key anti-apoptotic proteins, including B-cell lymphoma 2 (Bcl-2), B-cell lymphoma extra large (Bcl-xL), and X-linked inhibitor of apoptosis protein (XIAP). Additionally, the induction of apoptosis was linked to the suppression of the NF-κB signaling pathway [88]. The same research group further investigated the antitumor efficacy and mechanisms of action of bardoxolone methyl in human prostate cancer cell lines. Their results revealed that bardoxolone methyl inhibited cell growth and induced apoptosis in PC-3 and C4-2 cells at very low concentrations. The observed antitumor effects were linked to the suppression of key signaling proteins, including phosphorylated Akt (p-Akt), mTOR, and NF-κB, along with their downstream targets. Silencing of Akt triggered PC-3 cells to be more sensitive to bardoxolone methyl, while overexpression of Akt conferred resistance to the drug. Targeted silencing of Akt revealed that it did not regulate mTOR activation in PC-3 cells. However, silencing of mTOR enhanced the sensitivity of PC-3 cells to the growth-inhibitory effects of bardoxolone methyl, suggesting that Akt and mTOR are key molecular targets of bardoxolone methyl in prostate cancer cells. Notably, treatment with bardoxolone methyl was confirmed to be effective also in vivo, since it suppressed the growth of PC-3 xenografts in nude mice [89]. Indeed, PC-3 cells were injected in BALB/c nude mice either receiving bardoxolone methyl 10 μmol/kg or vehicle mixture only for 5 days a week over a 5-week period and tumors size was monitored. Tumors in the vehicle-only control group showed progressive growth over a 6-week period, with one mouse euthanized on day 39 due to tumor size. In contrast, treatment with bardoxolone methyl induced an initial increase in tumor size during the first two weeks, followed by a significant reduction in tumor volume, and after 5 weeks of treatment the average tumor size in the bardoxolone methyl group was notably smaller compared to the size at the initiation of treatment. After 50 days, tumors were harvested and weighed, revealing significantly lower tumor weights in the bardoxolone methyl-treated mice compared to the control group, suggesting a significant antitumor activity in vivo [89]. It has been reported that bardoxolone methyl can trigger the generation of ROS. Indeed, the inhibition of ROS production, either through the use of N-acetylcysteine (NAC) or by overexpressing antioxidant enzymes such as GPx and superoxide dismutase-1 (SOD-1), effectively prevented the bardoxolone methyl-induced apoptosis. Additionally, NAC was able to block the inhibition of constitutively active Akt, NF-κB, and mTOR by bardoxolone methyl. These results suggest that the inhibition of the PI3K/Akt/mTOR signaling pathway by bardoxolone methyl may occur in a ROS-dependent manner [90]. Khurana et al. explored the potential of bardoxolone-methyl in modulating androgen receptors expression in prostate cancer cells. At nanomolar concentrations, bardoxolone-methyl effectively downregulated androgen receptors expression in CWR22Rv1 cells at both the mRNA and protein levels. Mechanistic analysis revealed that short-term exposure (2 h) to bardoxolone-methyl resulted in increased ROS, whereas prolonged exposure (24 h) led to a reduction in ROS levels, accompanied by an upregulation of the antioxidant transcription factor Nrf2. NAC administration was able to reverse the androgen receptor-suppressive effects of bardoxolone-methyl. Furthermore, co-treatment of prostate cancer cells with bardoxolone-methyl boosted the efficacy of the anti-androgen enzalutamide (ENZ), as demonstrated by reduced cell viability, migration, and colony formation, suggesting that it could be a very useful drug in the management of prostate cancer [91].

### 4.4. Pancreatic Cancer

The anticancer properties and underlying mechanisms of bardoxolone methyl were investigated using human pancreatic cancer cell lines. The compound effectively suppressed the proliferation of both K-Ras mutant (MiaPaca2, Panc1, and Capan2) and wild-type K-Ras (BxPC3) pancreatic cancer cells at remarkably low concentrations. Its growth-inhibitory action was primarily linked to the induction of apoptosis, as demonstrated by elevated annexin-V-FITC staining, PARP-1 cleavage, and activation of caspase-3, -8, and -9. Bardoxolone methyl also caused a reduction in mitochondrial membrane potential and facilitated the release of cytochrome C. These antitumor effects were further associated with the downregulation of key pro-survival signaling molecules, including phosphorylated Akt (p-Akt), NF-κB, and mTOR, along with their downstream effectors such as phosphorylated Foxo3a (p-Foxo3a), p-S6 kinase 1 (p-S6K1), p-eIF-4E, and p-4E-BP1. Notably, silencing of Akt or mTOR using gene-specific siRNA enhanced the sensitivity of pancreatic cancer cells to bardoxolone methyl, indicating that these pathways are critical targets for its antiproliferative and pro-apoptotic activity [92]. Subsequently, it was further investigated the role of ROS in mediating apoptosis and inhibiting pro-survival signaling pathways, including Akt, NF-κB, and mTOR, by bardoxolone methyl in pancreatic cancer cells. Micromolar concentrations of bardoxolone methyl effectively inhibited cell proliferation and induced apoptosis in MiaPaCa-2 and Panc-1 pancreatic cancer cells. Interestingly, treatment with bardoxolone methyl led to the generation of hydrogen peroxide and superoxide anions. Pre-treatment with diphenylene iodonium, an NADPH oxidase inhibitor, or rotenone, an inhibitor of mitochondrial complex I, effectively suppressed the generation of reactive oxygen species (ROS). Additionally, the antiproliferative activity of bardoxolone methyl was completely negated by either N-acetylcysteine (NAC) or overexpression of antioxidant enzymes such as glutathione peroxidase (GPx) and superoxide dismutase 1 (SOD-1). NAC also prevented bardoxolone methyl-induced apoptosis, as evidenced by reduced PARP-1 cleavage, inhibition of caspase-3, -8, and -9 activation, preservation of mitochondrial membrane potential, and inhibition of cytochrome c release from mitochondria. Treatment with bardoxolone methyl led to the suppression of phosphorylated Akt (p-Akt), phosphorylated mTOR (p-mTOR), and NF-κB, while simultaneously enhancing Erk1/2 activation. Notably, NAC was able to counteract these effects, indicating that the modulation of these signaling pathways by bardoxolone methyl is dependent on ROS. Collectively, these results suggest that bardoxolone methyl’s antiproliferative and pro-apoptotic effects are driven by a mechanism reliant on reactive oxygen species [93]. In another study utilizing pancreatic cancer cells, it was reported that the inhibition of cell proliferation and induction of apoptosis mediated by the bardoxolone methyl is linked to a reduction in hTERT gene expression, decreased hTERT telomerase activity, and modulation of several proteins involved in regulating hTERT expression and activity. Moreover, altering the expression of hTERT, either by its silencing or overexpression, affected the sensitivity of pancreatic cancer cells to bardoxolone methyl, suggesting that hTERT is a critical target for bardoxolone methyl in pancreatic cancer cells [94]. In line with these results, treatment of MiaPaCa-2 and Panc-1 pancreatic cancer cell lines with bardoxolone methyl resulted in hydrogen peroxide and superoxide anions production and in a reduction in telomerase activity. Pretreating the cells with NAC or overexpressing GPx or SOD-1 effectively blocked the telomerase-inhibiting effects induced by bardoxolone methyl. Additionally, inhibiting ROS generation prevented the downregulation of hTERT gene expression, hTERT protein production, and the expression of several hTERT-regulatory proteins, such as p-Akt, NF-κB, Sp1, and c-Myc. Thus, the inhibition of telomerase activity by bardoxolone methyl is mediated via a ROS-dependent mechanism [95]. Jutooru et al. explored the in vivo anti-neoplastic efficacy of bardoxolone methyl using an orthotopic pancreatic cancer model, where L3.6pL pancreatic adenocarcinoma cells were directly injected into the pancreas of male thymic nude mice (8- to 12-week-old). Treatment with bardoxolone methyl 7.5 mg/kg/day started 7 days post-cell injection and lasted for 28 days. The results demonstrated that bardoxolone methyl treatment significantly reduced pancreatic tumors size compared to the control group. Furthermore, Western blot analysis of tumor lysates revealed a significant reduction in the expression of Sp1, Sp3, and Sp4 proteins in tumors from bardoxolone methyl-treated mice compared to the control group, while the levels of vascular endothelial growth factor (VEGF), cyclin D1, and survivin were significantly lower [96]. Gao et al. also investigated the therapeutic potential of bardoxolone methyl in pancreatic ductal adenocarcinoma. Exposure to bardoxolone methyl resulted in marked inhibition of cell proliferation and robust induction of apoptosis in pancreatic ductal adenocarcinoma cell lines and the cytotoxic effects were associated with downregulation of key survival signaling mediators, including p-Akt, NF-κB, and p-mTOR, implicating disruption of multiple oncogenic pathways. Subsequently, to further assess its antitumor efficacy, bardoxolone methyl was administered in vivo in heterotopic (subcutaneous) and orthotopic (pancreatic tail) xenograft models using cells harboring either mutant (MiaPaCa-2) or wild-type (BxPC-3) KRAS alleles. In the BxPC-3 xenograft model, bardoxolone methyl treatment led to a significant reduction in tumor volume, accompanied by diminished expression of p-Akt and p-mTOR within the tumor microenvironment. Moreover, in an orthotopic model simulating post-surgical recurrence, administration of bardoxolone methyl following partial tumor resection significantly extended animal survival, and this prolonged survival was attributed to the agent’s capacity to eliminate residual neoplastic cells upon surgery. Thus, these findings provided preclinical evidence that bardoxolone methyl possesses dual utility in pancreatic ductal adenocarcinoma management—both as a primary antitumor agent and as an adjuvant therapy to suppress post-operative disease recurrence through targeting of minimal residual disease [97].

### 4.5. Colorectal Cancer

Colorectal cancer the second leading cause of cancer-related deaths worldwide [98,99]. Gao et al. evaluated the apoptosis-inducing potential of bardoxolone methyl in colorectal cancer cell lines. Bardoxolone methyl administration at micromolar concentrations (1.25–10 µM) significantly suppressed cell growth and viability of colorectal cancer cells, and the primary mechanism of cell destruction was apoptosis, as indicated by the cleavage of PARP-1, activation of caspases-3, -8, and -9, and mitochondrial depolarization. The apoptosis induction by bardoxolone methyl was accompanied by the suppression of pro-survival signaling proteins, including Akt, NF-κB, and mTOR, as well as NF-κB-regulated anti-apoptotic proteins such as Bcl-2, Bcl-xL, Bad, and survivin, suggesting that bardoxolone methyl displays a good potential for therapeutical treatment for advanced, chemotherapy-refractory colorectal cancer [100]. A subsequent study provided an insight into the role of ROS formation following bardoxolone methyl treatment of colorectal cancer cell lines. The results revealed that while bardoxolone methyl effectively inhibits the growth of colorectal cancer cells, pretreatment with NAC completely abolished this growth inhibition. Indeed, bardoxolone methyl induced a massive ROS generation, which was prevented by NAC, diphenylene iodonium and rotenone. Moreover, apoptosis induction by bardoxolone methyl also was blocked by NAC which abolished also the inhibition of pro-survival signaling proteins, suggesting that bardoxolone methyl exerts potent anticancer effects in colorectal cancer cells through the generation of free radicals, which in turn leads to growth inhibition and apoptosis [101].

### 4.6. Ovarian Cancer

Among gynecologic tumors, ovarian cancer is that one with the higher mortality rate due to the late diagnosis and chemoresistance occurrence, which favors cancer relapse [102]. The efficacy of bardoxolone methyl was also investigated in human ovarian cancer cells. Treatment with bardoxolone methyl significantly inhibited the growth of ovarian cancer cells by increasing apoptosis rate. This effect on apoptosis was mediated by the inhibition of anti-apoptotic signaling proteins p-AKT, NF-κB, and p-mTOR. These results highlight the potent antiproliferative and pro-apoptotic effects of bardoxolone methyl in ovarian cancer cells, primarily through suppression of the AKT/NF-κB/mTOR signaling pathway, supporting its potential as a promising therapeutic candidate for ovarian cancer [103]. A subsequent study explored the role of ROS in the antitumor activity of bardoxolone methyl in OVCAR-5 and MDAH 2774 ovarian cancer cells. Treatment with bardoxolone methyl resulted in increased ROS generation, specifically hydrogen peroxide, and pre-treatment with the antioxidant NAC effectively blocked ROS generation and prevented the inhibitory effects of bardoxolone methyl on cell proliferation. Moreover, NAC pretreatment reversed the downregulation of the anti-apoptotic proteins p-AKT, p-mTOR, NF-κB, BCL-2, BCL-xL, c-IAP1 and survivin, suggesting that ROS play a key role in mediating the antiproliferative and apoptosis-inducing effects of bardoxolone methyl in ovarian cancer cells [104]. Duan et al. investigated the efficacy of bardoxolone methyl in ovarian and breast cancer. Their findings revealed that bardoxolone methyl effectively reduces IL-6 secretion in paclitaxel-resistant ovarian cancer cells and specifically inhibits the nuclear translocation of Stat3 induced by IL-6 or oncostatin M. Bardoxolone methyl treatment led to a significant reduction in the phosphorylation of Stat3, Jak2, and Src in ovarian and breast cancer cell lines exhibiting constitutive Stat3 activation. This suppression of the IL-6/Stat3 signaling cascade was linked to decreased expression of Stat3-regulated anti-apoptotic genes, such as Bcl-X(L), survivin, and Mcl-1. Apoptosis was induced following treatment, as indicated by PARP cleavage, the release of its fragments, and increased levels of the apoptotic marker CK18Asp396. Notable, bardoxolone methyl enhanced the cytotoxic effects of chemotherapeutics paclitaxel, in the paclitaxel-resistant ovarian cancer cell line OVCAR8(TR), and cisplatin, in the cisplatin-resistant ovarian cancer cell line A2780cp70, thus suggesting that bardoxolone methyl interferes with signaling through multiple kinases involved in the IL-6-Stat3 and Src pathways, likely by targeting several points within these signaling networks [105]. Qin et al. reported that in ovarian cancer bardoxolone methyl acts as a selective inhibitor of deubiquitinating enzyme ubiquitin-specific protease 7 (USP7), which is known to be a critical regulator in the oncogenic processes of numerous malignancies. Experimental data confirmed that USP7 is upregulated in ovarian cancer cells compared to non-malignant controls and genetic silencing of USP7 via RNA interference significantly impaired tumor cell proliferation both in vitro and in murine xenograft models. Target engagement was validated using cellular thermal shift assays (CETSA) and drug affinity responsive target stability (DARTS) assays, both of which confirmed direct binding of bardoxolone methyl to USP7 in a cellular environment. This interaction led to marked depletion of key USP7 substrates, including MDM2, MDMX, and UHRF1—proteins implicated in cell cycle regulation and epigenetic stability. Notably, systemic administration of bardoxolone methyl resulted in a substantial reduction in tumor burden in vivo, confirming its therapeutic potential [106].

### 4.7. Glioblastoma

The efficacy of bardoxolone methyl was also evaluated in glioblastoma (U87MG, U251MG) and neuroblastoma (SK-N-MC) cell lines, very aggressive forms of brain tumors that exhibit limited or weak responses to current anti-neoplastic treatments. Glioblastoma and neuroblastoma cell lines revealed to be very sensitive to bardoxolone methyl treatment, which was able to effectively inhibit cell proliferation at micromolar concentrations (2.5–10 µM). The primary mechanism of tumor cell destruction was apoptosis which was accompanied by the activation of caspases-3, -8, and -9, mitochondrial depolarization, the release of cytochrome c from mitochondria and downregulation of the anti-apoptotic proteins p-AKT, NF-κB, and Notch1, highlighting the potential of bardoxolone methyl for the treatment of aggressive brain tumors [107].

### 4.8. Osteosarcoma

Ryu et al. explored the efficacy of bardoxolone methyl in several multidrug-resistant osteosarcoma cell lines. Indeed, osteosarcoma is a highly aggressive malignancy with very limited pharmacological therapeutic options, and the activation of the STAT3 pathway is associated with tumor progression, cell survival, drug resistance, and adverse prognosis in this malignancy [108,109]. While multidrug-resistant osteosarcoma cell lines exhibit a constitutive activated Stat3 pathway with elevated levels of p-STAT3, treatment with bardoxolone methyl inhibited cell growth and induced apoptosis in osteosarcoma cells, significantly reducing the nuclear translocation and p-STAT3. This inhibition of the STAT3 pathway was associated with decreased expression of the anti-apoptotic genes Bcl-XL, Survivin, and MCL-1. Nonetheless, bardoxolone methyl treatment enhanced the cytotoxicity of doxorubicin in the osteosarcoma cell lines, thus highlighting its potential as a therapeutic agent, either alone or in combination with doxorubicin, for treating osteosarcoma and overcoming drug resistance [108].

Zou et al. explored whether bardoxolone methyl could activate the extrinsic, death receptor (DR)-mediated apoptotic pathway in human lung cancer cells. Their findings demonstrated that bardoxolone methyl not only triggered caspase-8 activation but also upregulated the expression of death receptors, particularly DR5, through a mechanism independent of p53. This upregulation enhanced TRAIL-induced apoptosis, regardless of the cell’s p53 status. Silencing DR5 using small interfering RNA reduced apoptosis caused by bardoxolone methyl alone and diminished the amplified apoptotic response when combined with TRAIL, indicating that DR5 induction is crucial for both bardoxolone methyl-induced and TRAIL-enhanced apoptosis. Additionally, bardoxolone methyl was found to rapidly activate c-Jun N-terminal kinase (JNK) prior to DR induction and caspase-8 activation. Inhibition of JNK signaling with the specific inhibitor SP600125 prevented bardoxolone methyl-induced JNK activation, DR expression, caspase-8 activation, and DNA fragmentation, suggesting that bardoxolone methyl promotes apoptosis in lung cancer cells via JNK-dependent upregulation of death receptors [110]. The mechanism by which bardoxolone methyl induces JNK-dependent DR5 expression was further investigated. Through analysis of DR5 promoter regions, it was observed that the CCAAT/enhancer binding protein homologous protein (CHOP) binding site is essential for bardoxolone methyl-induced transactivation of the DR5 gene. Consistent with this, bardoxolone methyl promoted both DR5 expression and CHOP upregulation. Inhibition of CHOP upregulation prevented the induction of DR5 expression by bardoxolone methyl, demonstrating that bardoxolone methyl induces CHOP-dependent DR5 upregulation. Additionally, the JNK inhibitor SP600125 blocked CHOP induction by bardoxolone methyl, suggesting that JNK activation is crucial for CHOP upregulation. Knockdown of CHOP significantly reduced bardoxolone methyl-induced apoptosis, confirming that CHOP plays a role in mediating bardoxolone methyl-induced cell death. Moreover, bardoxolone methyl elevated levels of Bip, phosphorylated eukaryotic translation initiation factor 2alpha, inositol-requiring kinase 1alpha, and activating transcription factor 4—key markers of endoplasmic reticulum (ER) stress. The use of salubrinal, an inhibitor of ER stress-induced apoptosis, not only inhibited JNK activation but also blocked CHOP and DR5 upregulation by bardoxolone methyl, protecting cells from apoptosis. These findings suggested that bardoxolone methyl induces ER stress, which in turns triggers JNK-mediated, CHOP-driven DR5 upregulation and apoptosis [111]. A subsequent study investigated how bardoxolone methyl affects the levels of c-FLIP, a key inhibitor of death receptor-mediated caspase-8 activation, and its role in bardoxolone methyl-induced apoptosis and the enhancement of TRAIL-induced apoptosis. Results showed that bardoxolone methyl promptly and significantly reduced c-FLIP levels in human lung cancer cell lines. Notably, the proteasome inhibitor MG132, but not the JNK inhibitor SP600125, prevented the decrease in c-FLIP levels induced by bardoxolone methyl. Furthermore, bardoxolone methyl increased the ubiquitination of c-FLIP, indicating that it induces c-FLIP degradation through a ubiquitin/proteasome-dependent pathway independent of JNK activation. Importantly, overexpression of c-FLIP prevented the activation of apoptosis induced by bardoxolone methyl, or from the combination of bardoxolone methyl and TRAIL. Conversely, silencing c-FLIP with siRNA increased the sensitivity of lung cancer cells to bardoxolone methyl, suggesting that the downregulation of c-FLIP plays a crucial role in both the initiation of apoptosis by bardoxolone methyl and the enhancement of TRAIL-induced apoptosis [112].

### 4.9. Oral Squamous Cell Carcinoma

The efficacy of bardoxolone methyl was explored also in oral squamous cell carcinoma (OSCC), the most common type of oral cancer which is associated with high morbidity and mortality also due to lack of effective therapies [113,114,115,116]. Hermann et al. evaluated the role of bardoxolone methyl in modulating radiotherapeutic responses and exerting cytotoxic effects on OSCC cell lines. Administration of bardoxolone methyl at nanomolar concentrations significantly suppressed OSCC cells proliferation and, upon exposure to an 8 Gy ionizing radiation dose, it markedly attenuated ROS accumulation in normal human epithelial keratinocytes, while enhanced oxidative stress in OSCC cells, even in the absence of radiation. Metabolic profiling revealed that bardoxolone methyl selectively augmented intracellular levels of reducing cofactors (NADH/NADPH) in malignant cells, suggesting a distinct shift in redox homeostasis. Furthermore, the expression of the antioxidant enzyme HO-1 was upregulated exclusively in normal human epithelial keratinocytes, with no corresponding increase observed in OSCC cells. Notably, colony formation assays indicated that clonogenic survival remained unaffected in normal keratinocytes following bardoxolone methyl treatment, whereas OSCC cells proliferation was almost abrogated. Thus, the authors concluded that bardoxolone methyl possesses both anti-neoplastic and radio-sensitizing properties specific to OSCC, while exerting minimal cytotoxicity on non-malignant epithelial cells, being a promising drug for selectively targeting malignant tissues [117]. Wang et al. investigated the cytotoxic and mechanistic effects of bardoxolone methyl in human esophageal squamous cell carcinoma (ESCC) cell lines Ec109 and KYSE70. Bardoxolone methyl treatment significantly inhibited cellular proliferation, induced G2/M phase cell cycle arrest, and promoted apoptosis in both cell lines. In particular, the G2/M arrest was associated with elevated levels of cyclin-dependent kinase inhibitor p21Waf1/Cip1 and tumor suppressor p53. Apoptotic induction was evidenced by the upregulation of pro-apoptotic Bax and the downregulation of anti-apoptotic proteins Bcl-2 and Bcl-xL, along with increased cleavage of caspase-9 and PARP. In addition to its pro-apoptotic activity, bardoxolone methyl triggered autophagy through inhibition of the phosphoinositide 3-kinase (PI3K)/mTOR pathway, a key signaling axis involved in cell survival and metabolic regulation. Cross-talk between autophagic and apoptotic responses was observed, indicating that these pathways may cooperate to mediate the cytotoxic effects of bardoxolone methyl. Bardoxolone methyl treatment also activated the Nrf2 signaling pathway, leading to enhanced detoxification of intracellular ROS, suggesting a dual role in redox homeostasis and cytoprotection, and attenuated invasive capacity and suppressed epithelial–mesenchymal transition (EMT) phenotypes in both Ec109 and KYSE70 cells. EMT modulation was characterized by decreased expression of the epithelial marker E-cadherin and upregulation of mesenchymal transcription factors including Snail, Slug, and ZEB1 (TCF-8). Notably, bardoxolone methyl reduced expression of key pluripotency and stemness-associated genes, including OCT4, Sox2, Nanog, and Bmi-1, which implies an inhibitory effect on the cancer stem-like cell population [118].

### 4.10. Other Malignancies

Nagaraj et al. investigated the effect of bardoxolone methyl across various tumor models, including MC38 colon carcinoma, Lewis lung carcinoma, and EL-4 thymoma in mice, alongside clinical samples from patients with renal cell carcinoma, soft tissue sarcoma, and pancreatic cancer treated with bardoxolone methyl in combination with gemcitabine. Results demonstrated that bardoxolone methyl, at nanomolar concentrations (25–100 nmol/L), effectively inhibited the immunosuppressive functions of myeloid-derived suppressor cells (MDSCs) in vitro without compromising their viability or influencing nitric oxide and arginase levels. Notably, the bardoxolone methyl reduced ROS levels in MDSCs but did not alter their proportion in spleen samples from tumor-bearing mice; however, it largely repressed their suppressive capacity. This immune-modulatory effect was independent of direct antitumor activity, although bardoxolone methyl effectively inhibited tumor growth in mice. Immune system involvement in this process was confirmed through studies with severe combined immunodeficient-beige mice, highlighting the capacity of the bardoxolone methyl to enhance cancer vaccine efficacy. In human patients with pancreatic cancer, bardoxolone methyl treatment did not affect MDSC counts in peripheral blood, yet it significantly bolstered the immune response. These findings suggest that bardoxolone methyl reverts MDSC-mediated immune suppression and potentiates immune responses, thus being a promising therapeutic molecule for cancer immunotherapy [119,120]. In the same study, peripheral blood mononuclear cells (PBMCs) from 19 patients (9 females and 10 males, ages 46–80) enrolled in a Phase I clinical trial RTA 402-C-0702, were analyzed. All participants had been diagnosed with either locally advanced (stage II-III) or metastatic (stage IV) pancreatic adenocarcinoma, and were not candidates for curative resection. Bardoxolone methyl was administered orally once daily for 21 consecutive days with a dosing regimen as follows: nine patients received 150 mg/day, two received 200 mg/day, six received 250 mg/day, and two received 300 mg/day. All patients also received intravenous gemcitabine (1000 mg/m^2^) on days 1, 8, and 15, beginning in the same week that bardoxolone methyl treatment started, and treatment cycles were repeated every 28 days. Treatment with bardoxolone methyl did not result in alterations in the quantity of MDSCs in peripheral blood, yet it significantly enhanced the immune response in these patients and no adverse effects attributable to the drug were reported [119].

Hong et al. [18] conducted a Phase I clinical trial to evaluate the DLTs, MTD, pharmacokinetics, pharmacodynamics, and antitumor efficacy of bardoxolone methyl. The compound was administered orally once daily for 21 consecutive days in a 28-day treatment cycle. Pharmacokinetic parameters were analyzed under both single-dose and steady-state conditions. Nrf2 pathway activation was assessed in PBMCs by measuring NAD(P)H:quinone oxidoreductase 1 (NQO1) mRNA expression, while tumor biopsies were examined via immunohistochemistry for markers related to inflammation, cell cycle progression, and apoptosis.

The study identified reversible grade 3 elevations in liver transaminases as the DLTs, with 900 mg/day established as the MTD. Clinically, one patient with mantle cell lymphoma achieved a complete response, and another with anaplastic thyroid carcinoma exhibited a partial response. Furthermore, NQO1 mRNA levels were elevated in PBMCs, while reductions in NF-κB and cyclin D1 expression were observed in tumor samples. An increase in estimated glomerular filtration rate (eGFR) was also noted, suggesting a potential renal benefit. Overall, bardoxolone methyl was well tolerated and showed promise for further clinical evaluation, particularly for its potential role in treating chronic kidney disease [18]. All the studies discussed in this review are reported in Table 1.

## 5. Limitations, Adverse Effects, and Safety Concerns

Despite the significant effects of bardoxolone methyl in anticancer therapy, some challenges facing the use of this drug should be taken into account. The concern regarding potential drug resistance or cellular adaptation due to long-term Nrf2 activation should be take into account, particularly given the transcription factor role in regulating antioxidant responses, detoxification, and cell survival pathways. Chronic activation of Nrf2, while protective in acute settings, has been associated in some contexts with undesirable outcomes such as enhanced survival of pre-malignant or malignant cells, chemoresistance, and metabolic reprogramming [121]. Prolonged upregulation of Nrf2 can lead to a sustained antioxidant environment that dampens reactive oxygen species (ROS)-mediated signaling, which is crucial for normal cellular functions such as apoptosis and immune surveillance. Furthermore, there is growing evidence that certain tumors exploit constitutive Nrf2 activation—often through mutations in KEAP1 or Nrf2—as a mechanism to evade chemotherapeutic stress, resist apoptosis, and sustain proliferation [122]. Therefore, in evaluating the therapeutic potential of Nrf2 activation, especially in chronic disease models, it is critical to assess the risk of cellular adaptation, including compensatory feedback mechanisms that may blunt the efficacy of treatment over time. Preclinical models and long-term treatment studies should include endpoints to monitor for signs of resistance, such as altered expression of Nrf2 target genes, cellular proliferation markers, or shifts in redox homeostasis. Additionally, intermittent dosing strategies or combination therapies may be considered to mitigate the risks of adaptation while preserving the cytoprotective benefits of Nrf2 activation. Overall, while Nrf2 remains a promising therapeutic target, its regulation must be approached with awareness of its dual role in health and disease to prevent unintended consequences of chronic activation.

Most of the studies conducted on bardoxolone methyl to date have utilized in vitro models, which involve testing in controlled laboratory environments using cell cultures. While these studies provide valuable insights into the potential mechanisms of action of the compound and biological effects, they do not fully replicate the complexity of a living organism. Therefore, further in vivo studies in animal models and well-designed clinical trials in humans are essential to validate the safety, efficacy, and therapeutic potential of bardoxolone methyl in a translational setting. Moreover, a better characterization of pharmacokinetics and bioavailability is required to achieve high efficacy and limited side effects.

Although bardoxolone methyl has demonstrated efficacy in preventing tumor progression in various in vivo models, its selectivity toward cancer cells over normal proliferative cells remains insufficiently characterized. The long-term effects of systemic bardoxolone administration and its potential impact on healthy tissues are still not fully understood. This raises important concerns about possible off-target effects and underscores the need for more selective therapeutic strategies. To mitigate these limitations, future research should focus on the development of targeted drug delivery systems that can preferentially direct bardoxolone methyl to tumor cells. Such approaches, including nanoparticle-based carriers or ligand-mediated targeting, hold promise for enhancing the therapeutic index of bardoxolone while minimizing toxicity to normal tissues.

Despite its promising therapeutic potential, bardoxolone methyl has raised several safety concerns, particularly in the context of long-term use. Clinical trials have reported adverse effects, including fluid retention, increased blood pressure, and cardiovascular events, which led to the early termination of some studies, such as the BEACON trial in patients with chronic kidney disease [17]. Given the therapeutic potential of bardoxolone methyl and the associated safety concerns, the rational design of novel derivatives or analogues based on its structure could offer a valuable strategy to overcome current limitations. By optimizing key functional groups or incorporating tumor-targeting features, it may be possible to improve selectivity, reduce systemic toxicity, and enhance the overall efficacy of treatment. Such efforts could lead to the development of next-generation Nrf2 modulators with a more favorable safety and pharmacological profile.

## 6. Conclusions and Perspectives

To the best of our knowledge, no comprehensive review in the last decade currently exists that consolidates and critically evaluates the available literature on the anticancer effects of bardoxolone methyl. This review aims to fill this gap by systematically integrating findings from both preclinical and clinical studies, highlighting mechanisms of action, therapeutic potential across various cancer types, and identifying existing limitations and areas for future research.

Numerous in vitro and in vivo studies have demonstrated that bardoxolone methyl holds both preventive and therapeutic promise, exhibiting antitumor effects across various cancer types primarily by modulating pathways that regulate cell proliferation and apoptosis. Despite these encouraging findings, further research is needed to identify new target proteins and clarify the intracellular signaling mechanisms involved. The establishment of specific endpoints and surrogate biomarkers to accurately measure therapeutic efficacy is also critical. Moreover, long-term epidemiological research and well-designed clinical trials with sufficiently large patient populations are necessary to validate these results. Future trials must carefully consider the safety concerns highlighted by the BEACON trial, which was halted due to an elevated risk of heart failure. Nevertheless, the collective in vitro and in vivo data reviewed here strongly support bardoxolone methyl as a promising candidate for both cancer prevention and treatment.

## Figures and Tables

**Table 1:** Anticancer effects of bardoxolone methyl.

Cancer Type	In Vivo/In Vitro	Anticancer Effects	Reference
Lung adenocarcinoma	In vivo (A/J mice + vinyl carbamate)	↓ 92% average tumor burden; ↓ number/size of tumors; ↓ high-grade tumors (1 vs. 36% in control)	[64]
Breast cancer (ER^-^)	In vivo (MMTV-neu mice)	Delayed the onset of ER^-^ tumors	[66]
Pancreatic cancer (KPC mouse model)	In vivo	↑ survival of mice by 3 to 4 weeks	[67]
Breast cancer (ER^-^)	In vivo	Delayed the initiation of tumor formation; ↑ overall survival by 5.2 weeks; ↓ tumor-associated macrophages into the mammary tissue; ↓ CXCL12 and CCL2 in the primary tumor cells and MMP9; ↓ proliferation, ↓ cyclin D1 expression; ↓ EGFR and STAT3 phosphorylation	[69]
Breast cancer (BRCA1-/p53^+/−^)	In vivo	Delayed tumor development; ↓ phosphorylation of ErbB2; ↓ cell proliferation; induced a G0/G1 cell cycle arrest	[70]
Chemoresistant breast cancer	In vitro/in vivo	In vitro: ↓ STAT3, Src, Akt, c-Myc levels; arrest cell cycle in G2-M phase; ↓ invasive growth of 4T1 cells no induction of apoptosis. In vivo: ↓ tumor progression	[71]
Breast cancer	In vitro	Induced extensive vacuolization originating from the endoplasmic reticulum triggers apoptotic death; ↑ calcium concentrations; ↑ ROS; ↓ c-FLIPL	[72]
Breast cancer	In vivo (PyMT mice)	↓ IL-10 and VEGF; ↑ TNF; ↓ CD206 and CD115 markers; ↓ CD4^+^ T; ↑ CD8^+^ T cells	[76]
Breast and lung cancer	In vitro (γ-irradiated cells)	↓ radiation-induced cytotoxicity; ↑ Nrf2; ↓ DNA damage	[77]
Breast cancer and mammary tumors	In vitro/in vivo	In vitro: ↑ lysosome-dependent ubiquitination and proteolytic degradation of LRP6 and FZD7; ↓ cytoplasmic β-catenin activation; ↓ Wnt target genes and CSC markers In vivo: impaired tumor progression; ↓ Wnt/β-catenin signaling inhibitor	[78]
Breast cancer	In vitro	Time-dependent decrease in cell migration; ↓ mitochondrial respiratory function; ↑ phosphorylation of AKT, DNA damage; ↓ cell proliferation. Changes in p65 phosphorylation and SOD2 expression, implying a role for non-canonical NF-κB signaling in bardoxolone methyl downstream effects	[80]
Prostate adenocarcinoma	In vivo (murine model)	↓ progression of preneoplastic lesions ↓ the metastatic process; ↓ TERT production and phosphorylation	[87]
Prostate cancer (LNCaP, PC-3 and DU145)	In vitro	↑ apoptosis in LNCaP and PC-3 cell lines; ↑ caspases-3, -8, and -9; ↓ anti-apoptotic proteins Bcl-2, Bcl-xL and XIAP; ↓ NF-κB signaling pathway	[88]
Prostate cancer	In vitro	↓ cell growth; ↑ apoptosis; ↓ p-Akt, mTOR, and NF-κB.	[89]
Prostate cancer	In vitro	↓ androgen receptors expression, cell viability, migration, and colony formation	[91]
Pancreatic cancer	In vitro	↓ growth of both K-Ras mutated (MiaPaca2, Panc1, and Capan2) and wild-type K-Ras (BxPC3) pancreatic cancer cells; ↑ apoptosis; triggered the loss of mitochondrial membrane potential and the release of cytochrome C; ↓ p-Akt, NF-κB, mTOR, p-Foxo3a, p-S6K1, p-eIF-4E, and p-4E-BP1	[92]
Pancreatic cancer	In vitro	↓ cell proliferation and ↑ apoptosis in MiaPaCa-2 and Panc-1 pancreatic cancer cells; ↓ p-Akt, p-mTOR, and NF-κB; ↑ Erk1/2	[95]
Pancreatic cancer	In vitro	↓ hTERT gene expression; ↓ hTERT telomerase activity; and modulation of several proteins involved in regulating hTERT expression and activity	[94]
Pancreatic cancer	In vitro	↑ hydrogen peroxide and superoxide anions; ↓ telomerase activity	[95]
Pancreatic cancer	In vivo (orthotopic pancreatic cancer model)	↓ pancreatic tumors size; ↓ expression of Sp1, Sp3, and Sp4 proteins; ↓ VEGF, cyclin D1, and surviving	[96]
Pancreatic ductal adenocarcinoma	In vitro/in vivo (heterotopic (subcutaneous) and orthotopic (pancreatic tail) xenograft models)	In vitro: ↓ inhibition of cell proliferation; ↑ apoptosis; ↓ p-Akt, NF-κB, and p-mTOR. In vivo: downregulation of key survival signaling mediators, including p-Akt, NF-κB, and p-mTOR In vivo: ↓tumor volume, expression of p-Akt and p-mTOR within the tumor microenvironment; ↑ survival	[97]
Colorectal cancer	In vitro	↓ cell growth and viability; ↑ apoptosis; cleavage of PARP-1; ↑ caspases-3, -8, and -9, and mitochondrial depolarization; ↓ Akt, NF-κB, and mTOR, NF-κB, Bcl-2, Bcl-xL, Bad, and survivin	[100]
Colorectal cancer	In vitro	↑ ROS generation	[101]
Ovarian cancer	In vitro	↓ growth of ovarian cancer cells; ↑ apoptosis; ↓ p-AKT, NF-κB, and p-mTOR	[103]
Ovarian cancer	In vitro	↑ ROS generation	[104]
Ovarian and breast cancer	In vitro	↓ IL-6 secretion in paclitaxel-resistant ovarian cancer cells; ↓ nuclear translocation of Stat3 induced by IL-6 or oncostatin; ↓ phosphorylation levels of Stat3, Jak2, and Src; ↓ Bcl-X(L), survivin, and Mcl-1, ↑ cleavage of PARP and the release of its fragments; ↑ cytotoxic effects of chemotherapeutics paclitaxel	[105]
Ovarian cancer	In vitro	↓ USP7; ↓ MDM2, MDMX, and UHRF1	[106]
Glioblastoma and neuroblastoma	In vitro	↓ cell proliferation; ↑ caspases-3, -8, and -9; mitochondrial depolarization; release of cytochrome c from mitochondria; ↓ p-AKT, NF-κB, and Notch1	[107]
Osteosarcoma	In vitro	↓ STAT3 pathway, ↓ p-STAT3, Bcl-XL, Survivin, MCL-1 expression; apoptosis induction; ↑ doxorubicin cytotoxicity	[108]
Lung Cancer	In vitro	Activation of DR5 via the JNK-CHOP pathway, caspase-8 activation, ER stress induction, c-FLIP degradation, enhanced TRAIL-induced apoptosis	[110,111,112]
Colon Carcinoma (MC38), Lung (LLC), Thymoma (EL-4)	In vivo (mouse models)	Reversal of MDSC-mediated immunosuppression, reduction in ROS, enhanced immune response, tumor growth inhibition	[119,120]
Pancreatic adenocarcinoma	In vivo (clinical samples)	Enhanced immune response in combination with gemcitabine, without affecting MDSC counts, well tolerated in patients	[119]
Mantle Cell Lymphoma, Anaplastic Thyroid Carcinoma	In vivo (Phase I trial)	One complete response (mantle cell lymphoma), one partial (thyroid carcinoma), reduced NF-κB and cyclin D1, increased NQO1 mRNA (Nrf2 activation), elevated eGFR	[18]
Oral Squamous Cell Carcinoma (OSCC)	In vitro	Cytotoxic and radiosensitizing effects, redox disruption, selective toxicity to cancer vs. normal cells, suppressed proliferation and clonogenic survival	[117]
Esophageal Squamous Cell Carcinoma (ESCC)	In vitro	G2/M arrest (↑ p21, p53), apoptosis (↑ Bax, ↓ Bcl-2/Bcl-xL), caspase-9 and PARP cleavage, autophagy via PI3K/mTOR inhibition, Nrf2 activation, EMT and stemness suppression	[118]

## Data Availability

No new data were created or analyzed in this study. Data sharing is not applicable.

## Abbreviations

The following abbreviations are used in this manuscript:

AMPK	AMP-activated protein kinase
ARE	antioxidant response element
Bcl-2	B-cell lymphoma 2
Bcl-xL	B-cell lymphoma extra large
bZip	basic leucine zipper
CDDO	2-cyano-3,12-dioxooleana-1,9-dien-28-oic acid
CETSA	cellular thermal shift assays
CHOP	CCAAT/enhancer binding protein homologous protein
CKD	chronic kidney disease
COX-2	inducible cyclooxygenase 2
CSC	cancer stem cell
Cul3	Cullin3
DARTS	drug affinity responsive target stability
DLTs	dose-limiting toxicities
DMF	dose-modifying factor
DVL2	Disheveled 2
eGFR	estimated glomerular filtration rate
EGFR	epidermal growth factor receptor
EMT	epithelial–mesenchymal transition
FZD7	Frizzled-7
GCLC	glutamate-cysteine ligase catalytic
GCLM	glutamate-cysteine ligase modifier
GPx	glutathione peroxidase
GSK3	glycogen synthase kinase 3
GST	glutathione S-transferase
HO-1	heme oxygenase-1
IKK	IκB kinase
IL-10	interleukin-10
IL-6	interleukin-6
iNOS	nitric oxide synthase
JAK	Janus Kinase
Keap1	Kelch-like ECH-associated protein 1
LC–MS/MS	liquid chromatography–tandem mass spectrometry
LRP6	lipoprotein receptor-related protein 6
Maf	musculoaponeurotic fibrosarcoma
MAPK	mitogen-activated protein kinase
MDSCs	myeloid-derived suppressor cells
MMP-9	matrix metalloproteinase-9
MMTV	mouse mammary tumor virus
MTD	maximum tolerated dose
mTORC1	mechanistic target of rapamycin complex 1
NAC	N-acetylcysteine
NQO1	NADPH dehydrogenase quinone 1
Nrf2	erythroid 2-related factor 2
p-4E-BP1	phosphorylated 4E-BP1
PBMCs	peripheral blood mononuclear cells
p-eIF-4E	phosphorylated eIF-4E
pErbB2	phosphorylated ErbB2
p-Foxo3a	Foxo3a
PI3K	phosphoinositide 3-kinase
p-S6K1	phosphorylated S6 kinase 1
PyMT	polyoma-middle T
Rbx1	Ring-box 1
ROS	reactive oxygen species
SDD	spray-dried dispersion
SOD-1	superoxide dismutase-1
STATs	Signal Transducers and Activators of Transcription
TERT	telomerase reverse transcriptase
TNF	tumor necrosis factor
VEGF	vascular endothelial growth factor
XIAP	X-linked inhibitor of apoptosis protein
